# Synthesis, Characterization and *In-vitro* Evaluation of Novel Naphthoquinone Derivatives and Related Imines: Identification of New Anticancer Leads

**Published:** 2019

**Authors:** Sima Golmakaniyoon, Vahid Reza Askari, Khalil Abnous, Afshin Zarghi, Razieh Ghodsi

**Affiliations:** a *Biotechnology Research Center, Mashhad University of Medical Sciences, Mashhad, Iran. *; b *Department of Medicinal Chemistry, School of Pharmacy, Mashhad University of Medical Sciences, Mashhad, Iran. *; c *Department of Pharmacology, Faculty of Medicine, Mashhad University of Medical Sciences, Mashhad, Iran.*; d *Pharmaceutical Research Center, Mashhad University of Medical Sciences, Mashhad, Iran. *; e *Department of Pharmaceutical Chemistry, School of Pharmacy, Shahid Beheshti University of Medical Sciences, Tehran, Iran.*

**Keywords:** Synthesis, 2-hydroxynaphthalene-1, 4-dione, Naphthoquinone, Bis naphthoquinone, Benzoxantene-6, 11-dione, Benzoacridine-5, 6-dione, Anticancer

## Abstract

Quinones such as 1,4-naphthoquinones are abundant in nature and naphthoquinone based natural products are known to possess anticancer activity. This pharmacophore is known to convey anticancer activity to some drugs such as streptonigrin, mitomycin A, *etc*. We synthesized and characterized different classes of naphthoquinone derivatives including bis naphthoquinone, 2-arylaminonaphthoquinone, benzoxantene-6,11-dione and benzoacridine-5,6-dione derivatives instead of the expected 2-hydroxy-3-(substituted phenyl(aryl amino)methyl)naphthalene-1,4-dione derivatives from the reaction of 2-hydroxy1,4-naphthoquinone (lawson) with different benzaldehydes and aryl amines. Benzoacridine-5,6-dione derivatives and related imines showed potent anti-breast cancer activity in MCF-7 cancer cells. The *in-vitro* results revealed that five compounds benzoacridinedione derivatives (**6b** and **7b**) and imines (**13**, **14** and **15**) by the IC_50_ range of 5.4-47.99 μM are the most potent anti-breast cancer structures.

## Introduction

**Figure 1:**
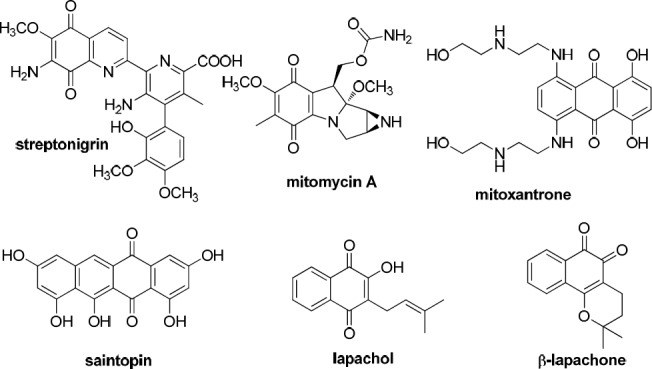
Chemical structures of known anticancer naphthoquinone based compounds

Quinones have been frequently exploited for the discovery of cellular mechanisms associated with cytotoxicity in various cancer cells. The redox properties of quinones can often induce apoptosis in cancer cells through oxidative stress induced by the *in-situ* creation of reactive oxygen species (ROS), while additional evidence proposes that some quinones can intercalate with DNA or inhibit proteins involved in DNA replication (1). Quinones such as 1,4-naphthoquinones are abundant in nature (1) and naphthoquinone based natural products are known to possess anticancer activity. This pharmacophore is known to convey anticancer activity to some drugs such as streptonigrin, mitomycin A,* etc* (2, 3). 1,4-Naphthoquinones are extensively distributed in nature and many well-known important anticancer drugs having a quinone moiety such as anthracyclines, mitoxantrones, and saintopin (Figure 1) have shown tremendous anticancer activity (4). 

The naturally occurring naphthoquinone lapachol is the most plentiful naphthoquinoidal compound isolated from the core of the trees of the family *Bignoniaceae*. This natural product has been widely studied due to its important biological activities, including antitumoral (5, 6). β-lapachone is the most favorable molecule of the lapachol group. It is cytotoxic to a variety of human cancer cells (7), which are naturally more prone to oxidative damage in comparison to normal cells (8). β-lapachone has been widely studied in recent years and is now in phase II clinical trials as a monotherapy or in combination with other antitumor drugs (9).

**Figure 2:**
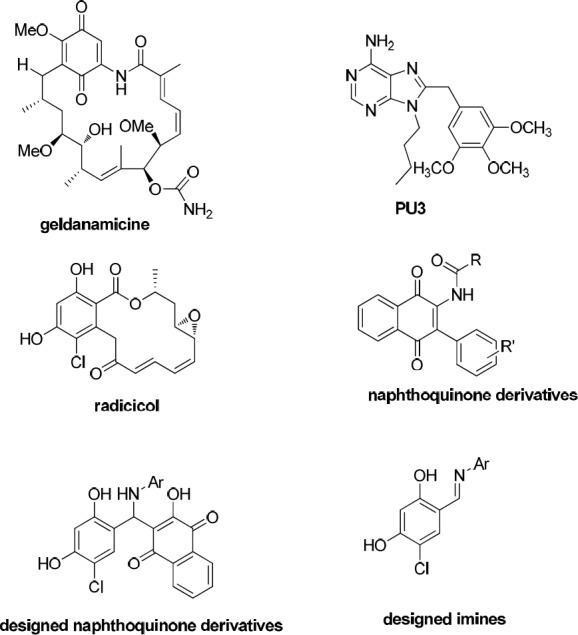
Chemical structures of known Hsp90 inhibitors and our designed compounds

On the other hand, the discovery of heat shock protein 90 (Hsp90) as the target of anticancer activity of geldanamycin (Figure 2) has been attracted much attention in inhibition of Hsp90 as a tactic for the treatment of cancer. This led to huge efforts to develop clinically practical small Hsp90 inhibitor molecules (10-12) with a wide structural diversity including purine-based analogues (PU3) (11), naphthoquinone based structures (13, 14), and resorcinol based structures (radicicol is a natural resorcinol derivative, Figure 2) (15, 16). 

In this study, we designed some 2-hydroxy-3-(substituted phenyl(aryl amino)methyl) naphthalene-1,4-dione derivatives possessing naphthoquinone and resorcinol rings as potential Hsp90 inhibitors. Furthermore, we designed related imines, which are lacking in naphthoquinone moiety, to investigate the role of naphthoquinone ring in biological activity of the designed naphthoquinone derivatives (Figure 2). We characterized the structures of the prepared products by ^1^H-NMR, ^13^C-NMR, Mass spectrometry, and CHN analysis. Surprisingly, what we separated was different from the expected2-hydroxy-3-(substituted phenyl (aryl amino) methyl) naphthalene-1, 4-dione derivatives. The synthesized compounds were evaluated for their cytotoxic activity towards MCF-7 and PC3 cancer cell lines to find new anticancer leads.

## Experimental

*General*

All chemicals, reagents, and solvents were purchased from Merck AG and Aldrich Chemical. Melting points were determined on a Thomas–Hoover capillary apparatus. Infrared spectra were taken on a Perkin Elmer Model 1420 spectrophotometer in KBr pellets. ^1^H and ^13^C-NMR spectra were recorded on a Bruker FT-300 and 400 (Bruker Biosciences, USA) instrument at 300 (and 400) MHz and 75 MHz respectively, in DMSO-*d*_6_ or CDCl_3_ as solvents. The chemical shifts are recorded in ppm relative to tetramethylsilane as internal standard. Coupling constant (*J*) values are in hertz (Hz) and spin multiples are given as s (singlet), d (double), t (triplet), q (quartet), and m (multiplet). Mass spectral data were recorded on a 6410 Agilent LCMS triple quadrupole mass spectrometer (LCMS) with an electrospray ionization (ESI) interface. 

*Synthesis of 3-chloro-2,4-dihydroxybenzaldehyde (* ***1b*** *)*

Pale yellow solid; IR (*v*_max_ cm^-1^) (KBr): 3321 (OH), 1662 (C=O); ^1^H NMR (300 MHz-CDCl3) δ = 11.93 (s, 1H, CHO), 9.65 (s, 1H, OH), 7.33-7.36 (d, 1H, 2-chlorobenzenediol, *J* = 9.6 Hz), 7.48-8.03 (s, 1H, OH), 6.63-6.66 (d, 1H, 2-chlorobenzenediol, *J* = 9.3 Hz). 

*Synthesis of 2-((1,4-dihydro-2-hydroxy-1,4-dioxonaphthalen-3-yl) (4-hydroxyphenyl) methyl)-3-hydroxynaphthalene-1,4-dione (* ***5*** *)*

A solution of amine **3a **(1 mmol) and aldehyde **1d** (1 mmol) was refluxed in ethanol for 3 h. Then, 1 mmol 2-hydroxy-1, 4-naphthoquinone, and 20 mol% InCl_3_ as catalyst were added to the mixture and put under reflux for an overnight. The resulted mixture was cooled down to room temperature and filtered. The precipitate was washed with a blend of ethanol-water (1:1 v/v ratio) and continued with ethanol lonely until the pure product was obtained. 

Dark brick red solid; m.p: 175-177 °C (same as reference (17, 18)); IR (*v*_max_ cm^-1^) (KBr): 3321 (OH), 1662 (C=O); ^1^H NMR (300 MHz-DMSO) δ = 6.33 (s, 1H, CH), 6.55-6.57 (d, 2H, 4-hydroxyphenyl, H_3_ & H_5_, *J* = 8.8 Hz), 6.91-6.93 (d, 2H, 4-hydroxyphenyl, H_2_ & H_6_, *J *= 8.4 Hz), 7.68- 7.72 (t, 2H, 3-hydroxynaphthalen-1,4-dione, H_7_, *J* = 8 Hz), 7.76- 7.80 (t, 2H, 3-hydroxynaphthalen-1,4-dione, H_6_, *J* = 8 Hz), 7.90- 7.92 (d, 2H, 3-hydroxynaphthalen-1,4-dione, H_8_, *J* = 8 Hz), 7.93-7.95 (d, 2H, 3-hydroxynaphthalen-1,4-dione, H_5_, *J* = 8.4 Hz), 9.01(s, 1H, OH), 9.08 (s, 1H, OH); LC-MS (ESI): 453.1 (M+1)^+^.

*Synthesis of 7-(4-hydroxyphenyl)-10,11-dihydrobenzo[h][1,4]dioxino[2,3-b]benzoacridine-5,6-dione* *(****6b****) *

1 mmol arylamine **3b** and 1 mmol benzaldehyde **1d** were solved in 4 mL ethanol. After 30 min stirring, 1 mmol naphthoquinone, 40 mol% InCl_3_ and 3 mL ethanol were added to the mixture reaction. The mixture was refluxed for an overnight. Subsequently, 80 mL water was added, resulting in the formation of fine precipitate. It was then filtered and the pure product was finally separated by preparative layer chromatography (eluent: chloroform/ethanol (60/6, v/v)). 

Dark orange solid; m.p: 205 °C (decomposed); IR (*v*_max_ cm^-1^) (KBr): 3368 (OH), 1662 (C=O); ^1^H NMR (300 MHz-DMSO) δ = 4.37-4.38 (s, 2H, OCH_2_), 4.43-4.45 (s, 2H, OCH_2_), 6.79 (s, 1H, benzodioxin), 6.90-6.93 (d, 2H, 4-hydroxyphenyl, H_3_ & H_5_, *J* = 8.4 Hz), 7.05-7.08 (d, 2H, 4-hydroxyphenyl, H_2_ & H_6_, *J *= 8.4 Hz), 7.55 (s, 1H, benzodioxin), 7.63-7.69 (dt, 1H, benzoacridinedione, H_2_, *J *= 17.7 Hz, *J *= 8.4 Hz), 7.87- 7.92 (dt, 1H, benzoacridinedione, H_3_, *J *= 16.5 Hz, *J *= 8.1 Hz), 7.99-8.03 (dd, 1H, benzoacridinedione, H_1_, *J *= 12 Hz, *J *= 4.2 Hz), 8.83-8.86 (d, 1H, benzoacridinedione, H_4_, *J *= 7.5 Hz), 9.65-9.75 (s, 1H, OH); ^13^C NMR (75 MHz-DMSO) δ = 64.4, 67.5, 112.53, 114.28, 115.60, 122.13, 124.18, 126.61, 127.6, 128.36, 128.90, 129.92, 131.10, 132.25, 132.57, 135.78, 137.88, 145.59, 150.06, 150.31, 151.92, 157.60, 180.22, 180.26; LC-MS (ESI): 410.1 (M+1)^+^. Anal. Calcd for C_25_H_17_NO_5_: C, 72.99; H, 4.16; N, 3.40. Found: C, 69.85; H, 3.98; N, 3.22.

*Synthesis of 7-(4-hydroxyphenyl)-8,9,10-trimethoxybenzo[c]acridine-5,6(7H,12H)-dione (* ***7b*** *)*

One mmol arylamine **3c** and 1 mmol benzalaldehyde **1d** were solved in 4 mL ethanol and refluxed for 3 h, then 1 mmol naphthoquinone and 40 mol% InCl_3_ were added to the resulted solution. After an overnight reflux, 60 mL water was added. The resulting precipitate was filtered and washed with hexane to give the pure product.

Violet solid; m.p: 209-211 °C; IR (*v*_max_ cm^-1^) (KBr): 3293 (OH), 1690 (C=O); ^1^H NMR (300 MHz-DMSO) δ = 3.53 (S, 3H, OCH_3_), 3.70 (s, 3H, OCH_3_), 3.85 (s, 3H, OCH_3_), 5.33 (s, 1H, CH), 6.57-6.59 (d, 2H, 4-Hydroxyphenyl, H_3_ & H_5_, *J *= 8.7 Hz), 6.98-7.01 (d, 2H, 4-Hydroxyphenyl, H_2_ & H_6_, *J *= 8.7 Hz), 7.04 (s, 1H), 7.61-7.66 (t, 1H, Benzoacridinedione H_2_, *J *= 7.5Hz), 7.83-7.88 (t, 1H, Benzoacridinedione, H_3_, *J *= 7.5 Hz), 7.94-7.97 (d, 1H, Benzoacridinedione, H_1_, *J *= 7.8 Hz), 8.3-8.32 (d, 1H, Benzoacridinedione, H_4_, *J *= 7.8 Hz), 9.14 (s, 1H, OH), 10.18 (s, 1H, NH) ; ^13^C NMR (75 MHz-DMSO) δ = 56.21, 60.76, 60.90, 97.55, 111.89, 112.76, 115.16, 124.03, 128.62, 129.12, 130.71, 130.97, 131.29, 131.81, 134.79, 137.89, 139.41, 145.09, 150.94, 152.92, 155.95, 172.49, 174.97, 180.32. LC-MS (ESI): 444.2 (M+1)^ +^. Anal. Calcd for C_26_H_21_NO_6_: C, 70.42; H, 4.77; N, 3.16. Found: C, 66.75; H, 4.59; N, 3.02.

*Synthesis of 2-((2,3-dihydrobenzo[b][1,4]dioxin-6-yl)amino) naphthalene-1,4-dione* *(****8****)*

All steps described in synthesis of product **6b** were done with appropriate aldehyde and amine, but the eluent for preparative layer chromatography was the combination of chloroform/ethanol (60/4.5 v/v). 

Violet solid; m.p: 165 °C (decomposed); IR (*v*_max_ cm^-1^) (KBr): 3321 (NH), 1676 (C=O); ^1^H NMR (300 MHz-DMSO) δ = 4.25 (s, 4H, OCH_2_CH_2_O), 5.96 (s, 1H), 6.85-6.93 (m, 3H, arom), 7.76-7.79 (dt, 1H, *J *= 7.2 Hz, *J *= 1.2 Hz), 7.82-7.87 (dt, 1H, *J *= 7.2 Hz, *J *= 1.2 Hz), 7.91-7.94 (dd, 1H, *J *= 7.8 Hz, *J *= 1.5 Hz), 8.02-8.05 (dd, 1H, *J *= 7.5 Hz, *J *= 1.2 Hz), 9.12 (s, 1H, NH); ^13^C NMR (75 MHz-DMSO) δ = 64.33, 64.42, 102.91, 112.49, 116.60, 118.04, 126.16, 126.46, 130.43, 130.71, 132.23, 133.37, 134.87, 141.77, 144.09, 145.40, 182.13, 183.80; LC-MS (ESI): 308.1 (M+1)^+^. Anal. Calcd for C_18_H_13_NO_4_: C, 70.35; H, 4.26; N, 4.56; Found: C, 68.39; H, 3.99; N, 4.43.

*General procedure for the synthesis of* ***9-12***

Appropriate amine and aldehyde (1 mmol of each) were refluxed in ethanol for 3 h. one mmol naphthoquinone and 20 mol% InCl_3_ were added to the mixture and after one overnight refluxing, 100 mL water was added and the resulted precipitate collected by filtration. The pure product was separated by preparative layer chromatography in appropriate eluent as described below:

Chloroform/ethanol (90/8 v/v) for compounds **9** and **10**, chloroform/ethanol (60/3 v/v) for compound **11** and chloroform/ethanol (60/10 v/v) for compound **12**. 

*3-hydroxy-12-(3-hydroxy-1,4-dioxo-1,4-dihydronaphthalen-2-yl)-6H-benzo[b]xanthene-6,11(12H)-dione* *(****9****)*

Red orange solid; m.p: 185 °C (decomposed); IR (*v*_max_ cm^-1^) (KBr): 3070 (OH), 1667, 1639 (C=O); ^1^H NMR (300 MHz-DMSO) δ = 5.65 (s, 1H, CH), 6.41-6.45 (m, 2H), 6.88 (s, 1H ), 7.01-7.04 (d, 1H, *J* = 9 Hz), 7.45- 7.50 (t, 1H, *J *= 9 Hz), 7.60-7.65 (t, 1H, *J *= 9 Hz), 7.69- 7.72 (d, 1H, *J *= 9 Hz), 7.77-7.87 (m, 4H), 8.02-8.05 (dd, 1H, *J *= 6.6, *J *= 2.4), 9.45 (s,1H); ^13^C NMR (75 MHz-DMSO) δ = 67.49, 102.62, 112.79, 123.45, 124.43, 125.20, 125.38, 125.66, 126.0, 126.09, 129.75, 130.69, 130.89, 131.54, 132.02, 133.88, 134.28, 134.69, 135.85, 139.67, 149.82, 151.54, 156.78, 178.74, 188.12, 183.58, 188.35; LC-MS (ESI): 451.1 (M+1)^+^. Anal. Calcd for C_27_H_14_O_7_: C, 72.00; H, 3.13. Found: C, 69.92; H, 3.28.

*2-(3,4,5-trimethoxyphenylamino) naphthalene-1,4-dione* *(****10****)*

Dark pink solid; m.p: 138-140 °C; IR (*v*_max_ cm^-1^) (KBr): 3298 (NH), 1681 (C=O); ^1^H NMR (300 MHz-DMSO) δ = 3.68 (s, 3H, OCH_3_), 3.78 (s, 6H, OCH_3_), 6.13 (s, 1H), 6.72 (s, 2H, trimethoxyphenyl), 7.77-7.82 (dt, 1H, *J *= 7.5 Hz, *J *= 1.5 Hz), 7.851-7.906 (dt, 1H, *J *= 7.5 Hz, *J* = 1.5Hz), 7.95-7.98 (dd, 1H, *J *= 7.5 Hz, *J *= 0.9 Hz), 8.06-8.09 (dd, 1H, *J *= 7.5 Hz, *J *= 0.9 Hz), 9.14 (s, 1H, NH); ^13^C NMR (75 MHz-DMSO) δ = 56.33, 61.04, 100.79, 103.43, 126.22, 126.52, 130.35, 132.36, 133.17, 133.28, 134.97, 136.13, 145.15, 153.98, 182.03, 183.81; LC-MS (ESI): 340.1 (M+1)^+^. Anal. Calcd for C_19_H_17_NO_5_: C, 67.25; H, 5.05; N, 4.13 Found: C, 65.33; H, 4.79; N, 3.99.

*2-(naphthalen-1-ylamino) naphthalene-1,4-dione (* ***11*** *)*

Dark red pink solid; M.p: 140-143 ˚C; IR (*v*_max_ cm^-1^) (KBr): 3303 (OH), 1672 (C=O); ^1^H NMR (300 MHz-DMSO) δ = 5.23 (s, 1H), 7.51-7.66 (m, 4H, arom), 7.79-8.14 (m, 7H, arom), 9.57 (s, 1H, OH); ^13^C NMR (75 MHz-DMSO) δ = 103.89, 121.75, 122.43, 125.58, 126.21, 126.47, 126.74, 126.99, 127.39, 128.76, 130.49, 132.33, 132.90, 133.35, 134.55, 134.91, 146.50, 182.17, 183.78; LC-MS (ESI): 300.1 (M+1)^+^. Anal. Calcd for C_20_H_13_NO_2_: C, 80.25; H, 4.38; N, 4.68; Found: C, 75.34; H, 4.29; N, 4.59.

*4-chloro-3-hydroxy-12-(3-hydroxy-1,4-dioxo-1,4-dihydronaphthalen-2-yl)-6H-benzo[b]xanthene-6,11(12H)-dione (* ***12*** *)*

Dark orange solid; m.p: 250 °C (decomposed); IR (*v*_max_ cm^-1^) (KBr): 3433 (OH), 1644 (C=O); ^1^H NMR (300 MHz-DMSO) δ = 5.69 (s, 1H, CH), 6.62-6.64 (d, 1H, 4-Clorophenyl, *J *= 8.4 Hz), 6.90 (s, 1H, OH), 6.95-6.98 (d, 1H, 4-Clorophenyl, *J *= 8.7 Hz), 7.48-7.50 (t, 1H, *J *= 7.2 Hz), 7.63-7.72 (t, 1H, *J *= 7.2 Hz), 7.80-7.81 (d, 1H, *J *= 3.6 Hz), 7.82-7.86 (m, 4H, arom), 8.03-8.06 (dd, 1H, *J *= 7.2 Hz, *J *= 2.4 Hz), 10.15 (s, 1H, OH); ^13^C NMR (75 MHz-DMSO) δ = 67.49, 107.56, 112.78, 117.83, 123.17, 124.46, 125.24, 125.66, 126.04, 126.13, 127.09, 130.69, 130.92, 131.61, 131.96, 134.01, 134.21, 134.70, 135.85, 139.67, 146.02, 151.17, 152.86, 1169.47, 178.28, 183.51, 187.80; LC-MS (ESI): 485.1 (M+1)^+^. Anal. Calcd for C_27_H_13_ClO_7_: C, 66.89; H, 2.7. Found: C, 61.35; H, 2.57.

*General procedure for the synthesis of imines (* ***13-15*** *)*

Appropriate aldehyde (1 mmol) and amine (1 mmol) were solved in 4 mL ethanol. The reaction condition was 2 h stirring for imine **13** and an overnight refluxing for imine **14** and **15**. Then the reaction mixture was allowed to cool down to room temperature, filtered, and washed sufficiently with ethanol to give the desired pure product. 

*(E)-2-chloro-4-(((2,3-dihydrobenzo[b][1,4]dioxin-6-yl)imino)methyl)benzene-1,3-diol (* ***13*** *)*

Yellow solid; m.p: 194-196 °C; IR (*v*_max_ cm^-1^) (KBr): 3437 (OH), 1611 (C=N); ^1^H NMR (300 MHz-DMSO) δ = 4.27 (s, 4H, OCH_2_CH_2_O), 6.53-6.60 (m, 2H, benzodioxine, H_4,5_), 6.65-6.68 (d, 1H, 2-chlorobenzenediol, *J *= 9 Hz), 6.94 (s, 1H, benzodioxine, H_2_), 7.53-7.56 (d, 1H, 2-chlorobenzenediol, *J *= 9Hz), 8.83 (s, 1H, CH), 9.80 (s, 1H, OH), 15 (s, 1H, OH); LC-MS (ESI): 306.1 (M+1)^+^. Anal. Calcd for C_15_H_12_ClNO_4_: C, 58.93; H, 3.96; N, 4.58. Found: C, 55.96; H, 3.77; N, 4.26.

*(E)-2-chloro-4-(((3,4,5-trimethoxyphenyl)imino)methyl)benzene-1,3-diol (* ***14*** *)*

Yellow solid; M.p: 205-208 °C; IR (*v*_max_ cm^-1^) (KBr): 3052 (OH), 1597.7 (C=N); ^1^H NMR (300 MHz-DMSO) δ = 3.70 (s, 3H, OCH_3_), 3.85 (s, 6H, OCH_3_), 6.56-6.59 (d, 1H, 2-chlorobenzenediol, *J *= 9Hz), 6.83 (s, 2H, trimethoxyphenyl), 7.34-7.37 (d, 1H, 2-chlorobenzenediol, *J *= 9 Hz), 8.92-8.93 (s, 1H, CH), 11.02 (s, 1H, OH), 15.04 (s, 1H, OH); LC-MS (ESI): 338.1 (M+1)^+^. Anal. Calcd for C_16_H_16_ClNO_5_: C, 56.90; H, 4.77; N, 4.15. Found: C, 54.90; H, 4.57; N, 4.04. 

*(E)-2-chloro-4-((naphthalen-1-ylimino)methyl)benzene-1,3-diol (* ***15*** *)*

Yellow solid; M.p: 185-189 °C; IR (*v*_max_ cm^-1^) (KBr): 3047.4 (OH), 1611.6 (-C=N-); ^1^H NMR (300 MHz-DMSO) δ = 6.64-6.67 (d, 1H, 2-chlorobenzenediol, *J *= 9.6 Hz), 7.48-8.03 (m, 7H, arom), 8.17-8.20 (d, 1H, 2-chlorobenzenediol, *J *= 9.3 Hz), 9.00 (s, 1H, CH), 11.15 (s, 1H, OH), 15.09 (s, 1H, OH); LC-MS (ESI): 298.1 (M+1)^+^. Anal. Calcd for C_17_H_12_ClNO_2_: C, 68.58; H, 4.06; N, 4.70. Found: C, 64.90; H, 4.57; N, 4.54.

*Cytotoxicity assay*

*General procedure*

The MTT (3-[4, 5-dimethylthiazol-2-yl]-2,5-diphenyl tetrazolium bromide) based assay was carried out by seeding 5000 cancer cells per 180 µL RPMI complete culture medium in each well of 96-well culture plates (19, 20). The day after seeding, culture medium was replaced with medium containing standard anti-tumor agent doxorubicine as well as different concentrations of newly synthesized compounds and RPMI control (no drug). The cells were then incubated at 37 ºC in 5% CO_2_ incubator for 48 h. Then 25 µL of MTT solution (4 mg mL^-1^) was added to each well and further incubated at 37 ºC for 3 h. At the end of incubation, formazan crystals were dissolved in 100 µL of DMSO and plates were read in a plate reader (Synergy H4, USA) at 540 nm. This experiment was performed in triplicate determination each time

## Results and Discussion

*Chemistry*

**Scheme 1:**
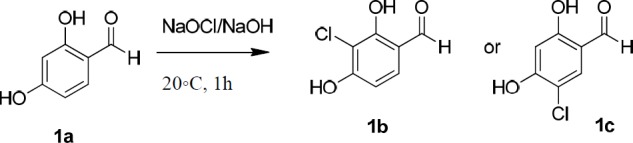
Synthesis of 3-chloro-2,4-dihydroxybenzaldehyde (**1b**)

One of the required substrate, 5-chloro-2,4-dihydroxy benzaldehyde (**1c**), was synthesized following a similar procedure described by Gupta *et al.* (21). Chlorination of the aromatic ring of **1a** was carried out using sodium hypochlorite solution in the basic aqueous medium (Scheme 1). But, (the observation of two doublet signals related to the two vicinal aromatic hydrogens instead of two singlet signals in ^1^H-NMR spectrum, supports that the achieved aldehyde is 3-chloro-2,4-dihydroxybenzaldehyde (**1b**)). The revealed ^1^H-NMR data as well as those pertaining to the reported structures possessing this aldehyde moiety in this work, conformed 3-chloro-2,4-dihydroxybenzaldehyde (**1b**) instead; emerging two doublet signals related to the two vicinal aromatic hydrogens instead of two singlet signals. We used 2,4-dihydroxybenzaldehyde (**1a**), 3-chloro-2,4-dihydroxybenzaldehyde (**1b**), and 4-hydroxybenzaldehyde (**1d**) as the starting materials in the next reaction.

**Scheme 2:**
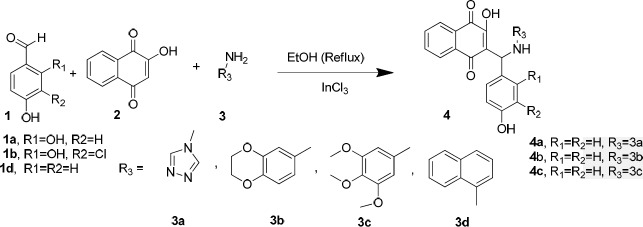
The starting materials (**1-3**) and some of the expected products (**4**)

As illustrated in Scheme 2, in order to achieve the synthesis of the desired compounds, Mannich reaction was employed in the presence of indium chloride as a catalyst in ethanol under reflux according to the reported procedure (22).

**Scheme 3:**
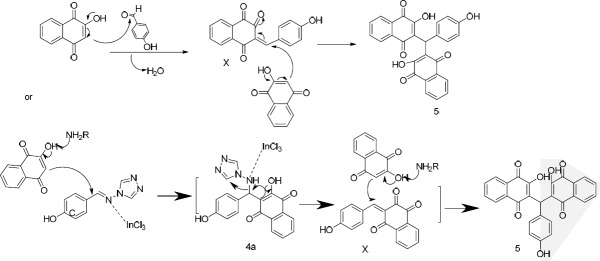
Proposed mechanisms for the formation of bisnaphtoquinone **5**

In order to synthesize **4a **(Scheme 2), a reaction of 4-hydroxybenzaldehyde (**1d**), 2-hydroxynaphthalene-1,4-dione (**2**), and4H-1,2,4-triazol-4-amine (**3a**) was carried out in the presence of InCl_3_ as a catalyst in ethanol under reflux. The obtained product was a bis-naphthoquinone analogue identifiedas 3,3′-(4-hydroxyphenylmethylene)bis(2-hydroxynaphthalene-1,4-dione) (**5**, entry 1, Table 1). In another attempt to synthesize **4a**, the reported method using *p*-TSA as the catalyst was carried out (23), but the main product was **5** instead. The formation of this product probably occurs through nucleophilic addition of 2-hydroxy-1,4-naphthoquinone to the resulting intermediate X (Scheme 3) in the presence of indium chloride. We did not investigate the exact mechanism of this reaction but a rational possibility mechanism for the formation of the3,3′-(4-hydroxyphenylmethylene)bis(2-hydroxynaphthalene-1,4-dione) (**5**) is shown in Scheme 3.

**Table 1:** Reactions and obtained or possible isomeric products

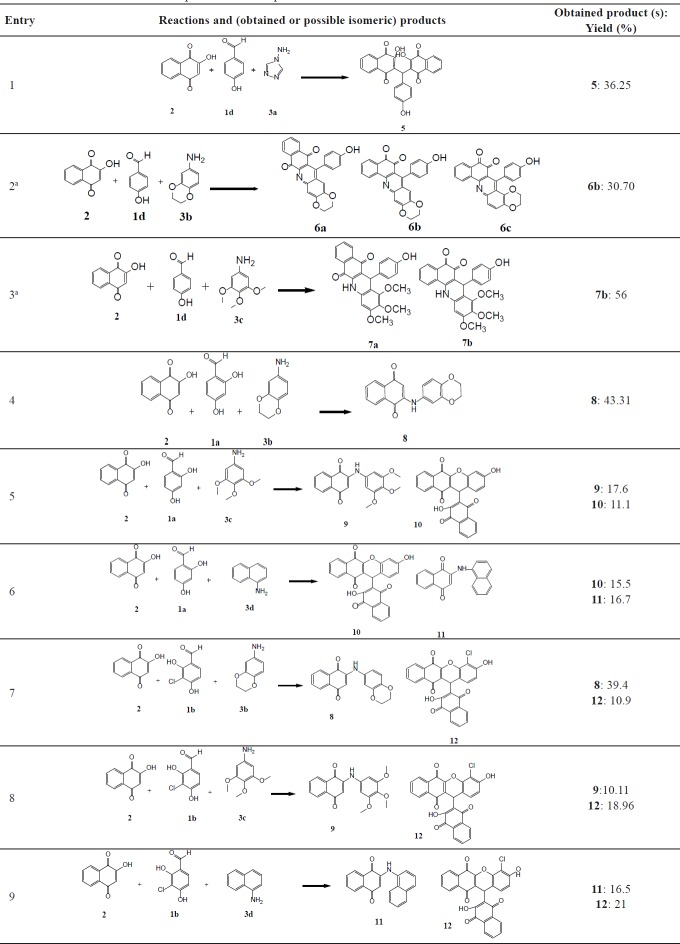

a Reactions with possible isomeric products.

**Table 2:** The *in-vitro *antiproliferative activities of compounds, doxorubicin and 17-AAG against MCF-7 (human breast cancer cells) and PC3 (human prostate cancer cells)

**Compound**	**MCF-7 IC** **50 ** **(μM)**	**PC3 Cell viability (%)** b
**5**	>100	64.36 ± 2.43
**6b**	47.99 ± 3.11	57.31 ± 3.11
**7b**	5.4 ± 1.49	59.42 ± 2.31
**8**	>100	67.92 ± 3.45
9	131.28 ± 2.95	50.36 ± 1.67
**10**	>100	62.65 ± 2.21
**12**	>100	62.49 ± 1.98
**13**	17.54 ± 2.15	56.36 ± 1.69
**14**	12.8 ± 2.31	49.63 ± 2.31
**15**	5.69 ± 1.71	51.65 ± 1.16
Doxorubicin	0.25 ± 0.04	0.64 ± 0.1 (IC50 (μM))
17-AAG	0.19 ± 0.08	Not determined

a Compound concentration required to inhibit tumor cell proliferation by 50%. Data are presented as the mean ± SEM from the dose− response curves of three independent experiments.

b The mean cell viability (%) ± SEM from the dose−response curves of three independent experiments at 100 μM concentration.

**Scheme 4:**
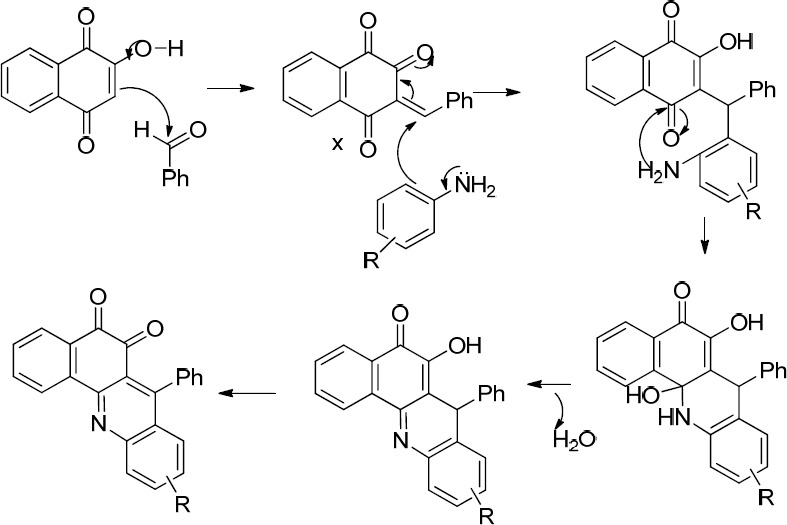
Proposed mechanism for the formation of **6b** and **7b**

**Scheme 5:**
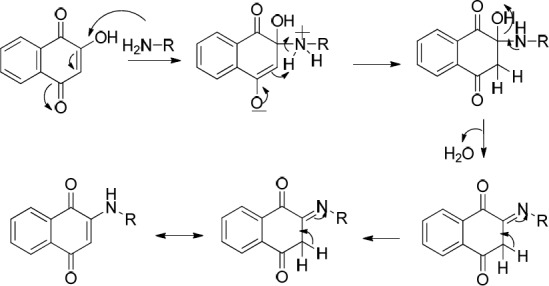
Proposed mechanism for the formation of 2-arylaminonaphthoquinones (**8**, **9** and **11**)

**Scheme 6:**
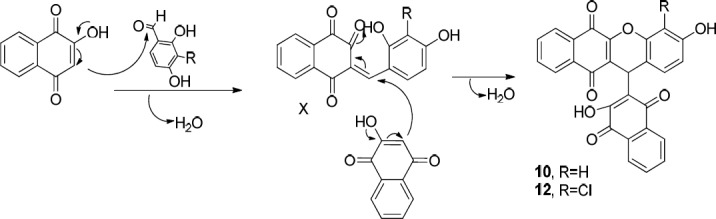
Proposed mechanism for the formation of benzoxanthene-6,11-dione derivatives (**10 **and **12**)

**Scheme 7:**
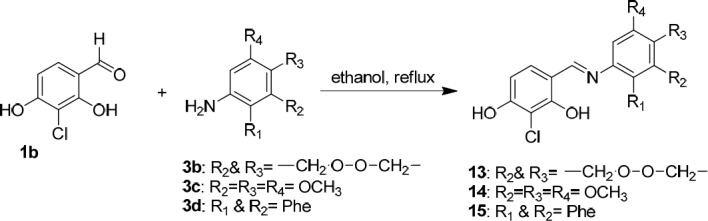
Synthesis of novel Schiff bases of 3-chloro-2,4-dihydroxybenzaldehyde (**1b**)

As shown in Scheme 2, the expected product from the reaction using **1d**, **2,** and **3b** as the starting materials was **4b** and in the case of applying **1d**, **2,** and amine **3c, **the formation of **4c** was expected. However, one aromatic hydrogen loss in the ^1^H-NMR data did not accommodate with these structures. This evidence suggests the formation of compounds **6a** and **7a **(entries 2 and 3 in Table 1) in which a new ring is established. On the other hand, the pattern of signals related to the aromatic hydrogens of naphthoquinone in ^1^H-NMR spectra demonstrated a noticeable change compared to the other structures containing naphthoquinone: further deshielding of one of the aromatic hydrogen in comparison with the other three ones suggests the structures **6b** and **7b.** As a matter of fact, the presence of two carbonyl groups in an *ortho* arrangement in the vicinity of one H atom of naphthoquinone ring, leads to shift its signal to the downfield to a greater degree. Moreover, the very two close carbonyl resonances at 180.22 and 180.26 ppm, which are the very adjacent chemical shifts for the carbonyl ^13^C-NMR signals, propose an *ortho*-quinone moiety which is seen in the structure of **6b**. A similar mechanism to that of Zhang *et al.* reported (24), describing the formation of benzoacridine-5,6-dione derivatives, is given in Scheme 4. Recently, the synthesis of different benzo[h]quinoline-5,6-dione derivatives and related compounds using 2-hydroxynaphthalene-1,4-dione have been reported (2, 24-28). It is noteworthy that the reaction in entry 2 in Table 1 could be accomplished in two probable cyclization pathways leading to give two different products; angular (14-(4-hydroxyphenyl)-2,3-dihydrobenzo[h][1,4]dioxino[2,3-a]acridine-12,13-dione, **6c**), and linear(7-(4-hydroxyphenyl)-10,11-dihydrobenzo[h][1,4]dioxino[2,3-b]benzoacridine-5,6-dione,**6b**). The ^1^H-NMR signals proposed compound **6b **as the only product of this reaction, and the angular analogue **6c** was not obtained. A possible reason for the formation of **6b** can be attributed to the less steric hindrance (29). On the whole, it can be concluded that the formation of bis naphthoquinone and benzoacridine-5,6-dione derivatives is as a result of competition between naphthoquinone and aryl amine in a nucleophilic attack to the intermediate X(3-(4-hydroxybenzylidene)naphthalene-1,2,4(3H)-trione), see entries 1-3 in Table 1).

Another two classes of naphthoquinone derivatives achieved in the case of using *ortho* hydroxyl-containing aldehydes **1a** and **1b**, are 2-arylaminonaphthoquinone and benzoxanthene-6,11-dione derivatives (see entries 4-9 in Table 1). As amine **3b** participated in the reaction, the product is merely the derivative of 2-arylaminonaphthoquinone **8 **(see entry 4 in Table 1). In the case of applying amine **3d** and **3c**, the other novel product, benzoxanthene dione derivative (**10** and **12**) was obtained, as well. A reasonable possibility for the formation of 2-arylaminonaphthoquinones (**8**, **9** and **11)** is shown in Scheme 5.

The probable initial formation of hydrogen bonding between hydroxyl group of naphthoquinone and amino group makes the C2 of lawson more electrophilic which facilitates the reaction through Michael addition mechanism (30). The probable sequence of steps leading to formation of benzoxanthenedione derivatives is given in Scheme 6.

First, the hydroxyl group of naphthoquinone is deprotonated by the amine favoring the nucleophilic attack of naphthoquinone into the aldehyde and the formation of intermediate X. Afterwards, subsequent Michael addition of naphthoquinone to the intermediate X and eliminating one water molecule afforded the corresponding products **10** and **12**. As a matter of fact, the formation of benzoxanthenedione products is due to the *ortho* hydroxyl group of aldehyde which leads to the conversion of bisnaphthoquinone (a symmetric system) into benzoxanthenedione (nonsymmetric system) derivatives. This symmetric and nonsymmetric system can obviously be seen in the ^1^H-NMR patterns of the aromatic hydrogens of two lawson molecules in the products (compare ^1^H-NMR data of **5 **as a symmetric bisnaphthoquinone analogue with **10 **as a nonsymmetric bisnaphthoquinone analogue). Furthermore, Pelageev *et al.* (31) reported the formation of 7,10-dihydroxy-12H-benzo[b]xanthene-6,11-dione derivatives resulted from the reaction of hydroxy naphthazarins and o-vanillin containing a hydroxyl group at position 2 under mild acid catalysis (acid catalyzed condition). On the other hand, the formation of benzoacridine-5,6-dione derivatives (compare the obtained products of entries 2 and 3 with those of entries 4-9 in Table 1) through the reaction of benzaldehydes possessing hydroxyl group in *ortho* position (**1a** and **1b**) with 2-hydroxy 1,4-naphthoquinone **2** and aryl amines (**3b** and **3c**) did not occur. This can be most probably due to the more electron donating character of **1a** and **1b** in comparison to **1d** and can be attributed to the mechanism of formation of benzoacridine-5,6-dione derivatives (see Scheme 4). 

We also synthesized novel Schiff bases of 3-chloro-2,4-dihydroxybenzaldehyde possessing resorcinol ring (**13**, **14** and **15**) in ethanol using 3-chloro-2,4-dihydroxybenzaldehyde (**1b**), 2,3-dihydrobenzo[b][1,4]dioxin-6-amine (**3b**), 3,4,5-trimethoxyaniline (**3c**), and naphthalen-1-amine (**3d**) as the starting materials (Scheme 7).

*In-vitro cytotoxic effects*

The MTT assay was employed for the assessment of the cytotoxicity of prepared compounds to find new lead compounds in terms of antitumor activity.

All the synthesized compounds including naphthoquinone derivatives (bis naphthoquinone, 2-arylaminonaphthoquinone, benzoxantene-6,11-dione, benzoacridine-5,6-dione derivatives) and resorcinol-containing imines (**13**, **14** and **15**) were evaluated for their antiprolifrative activity against two cancer cell lines including PC3 (human prostate cancer cell lines) and MCF-7 (human prostate cancer cell lines) using doxorubicin as a positive control (Table 2). We can classify our newly synthesized compounds in five classes including Bis naphthoquinone, 2-arylaminonaphthoquinone, benzoxantene-6,11-dione,benzoacridine-5,6-dione derivatives, and imines. Two classes of compounds including benzoacridine-5, 6-dione derivatives, and imines (**6b**, **7b**, **13**, **14** and **15**) showed strong antiproliferative activity in MCF-7 cells by the IC_50_ range of 5.4-47.99 μM. The high toxicity of compounds **6b** (IC_50 _= 47.99 μM) and **7b **(IC_50 _= 5.4 μM) is likely due to naphthalene-1,2-dione moiety which is responsible for the cytotoxicity of beta-lapachone (*ortho*-naphthoquinone with potential antineoplastic and radiosensitizing activity). Moreover, the 9-folded anticancer effect of compound **7b** in comparison with that of compound **6b** can be attributed to two factors: the presence of trimethoxy groups and the aromaticity of pyridine ring. According to the literature describing the synthesis of aza-podophyllotoxin analogues (32), the planar ring in comparison to nonplanar one could result in the reduction of anticancer activity. 

Various Schiff bases were reported as Hsp90 inhibitors and anticancer agents (16, 21, 31 and 34) and herein, three novel Schiff bases of 3-chloro-2, 4-dihydroxybenzaldehyde (**13**, **14** and **15**) also showed high cytotoxicity in MCF-7 cells. All three imines are possessing resorcinol ring which is an essential pharmacophore for inhibition of Hsp90. Therefore, their anticancer effects might be attributed to their ability to inhibit Hsp90.

2-arylaminonaphthoquinone derivatives did not exhibit significant cytotoxicity, with the exeption of compound **9 **which showed moderate cytotoxicity in MCF-7 cells (IC_50 _= 131.28 μM). Whereas various 2-arylaminonaphthoquinones and related compounds have been reported possessing cytotoxic activity with different mechanisms (35-43).

Bis naphthoquinone (**5**) and benzoxantene-6,11-dione (**10** and **12**) derivatives which are all possessing 1,4-naphthoquinone moiety, did not exhibit significant cytotoxicity. One possible reason could be attributed to their high polarity leading to poor permeability to the lipophilic cell membranes. Generally our compounds did not show significant cytotoxic effects on PC3 cells in the concentration below 100 µM. Overall, benzoacridine-5,6-dione derivatives and imines (**6b**, **7b**, **13**, **14** and **15**) showed stronger cytotoxic effects on PC3 cells compared to the other compounds. Benzoacridine-5,6-dione derivatives (**6b** and **7b**) proved to be promising cytotoxic agents and have encouraged further evaluation of this scaffold. Additional work is in progress in an attempt to find more potent antitumor agents.

## Conclusion

In summary, we synthesized and characterized novel naphthoquinone derivatives which can be classified in four different classes including bis naphthoquinone, 2-arylaminonaphthoquinone, benzoxantene-6,11-dione and benzoacridine-5,6-dione derivatives. Lawson has been used as the starting material for the synthesis of a variety of biologically active compounds and materials with significant properties. In organic synthesis, it has been used in many reactions (17) and to the best of our knowledge, it is the first report of characterization of four different scaffolds from the reaction of lawson with different arylamines and benzaldehydes. On the whole, it can be concluded that the formation of bis naphthoquinone and benzoacridine-5,6-dione derivatives is as a result of competition between naphthoquinone and aryl amine in a nucleophilic attack to the intermediate X (see the products of entries 1-3 in Table 1). The formation of benzoxanthenedione products is due to the *ortho* hydroxyl group of aldehyde which leads to the conversion of bisnaphthoquinone (a symmetric system) into benzoxanthene dione (nonsymmetric system) derivatives. We have studied the *in-vitro* anti-cancer activity of these compounds against MCF-7 and PC3 cell lines by MTT test. The *in-vitro* results revealed that five compounds benzoacridinedione derivatives (**6b** and **7b**) and imine (**13**, **14** and **15**) by the IC_50_ range of 5.4-47.99 μM are the most potent antibreast cancer structures. 
